# Musicians are more consistent: Gestural cross-modal mappings of pitch, loudness and tempo in real-time

**DOI:** 10.3389/fpsyg.2014.00789

**Published:** 2014-07-28

**Authors:** Mats B. Küssner, Dan Tidhar, Helen M. Prior, Daniel Leech-Wilkinson

**Affiliations:** Department of Music, King's College LondonLondon, UK

**Keywords:** cross-modal mappings, gesture, embodied music cognition, musical training, real-time mappings

## Abstract

Cross-modal mappings of auditory stimuli reveal valuable insights into how humans make sense of sound and music. Whereas researchers have investigated cross-modal mappings of sound features varied in isolation within paradigms such as speeded classification and forced-choice matching tasks, investigations of representations of concurrently varied sound features (e.g., pitch, loudness and tempo) with overt gestures—accounting for the intrinsic link between movement and sound—are scant. To explore the role of bodily gestures in cross-modal mappings of auditory stimuli we asked 64 musically trained and untrained participants to represent pure tones—continually sounding and concurrently varied in pitch, loudness and tempo—with gestures while the sound stimuli were played. We hypothesized musical training to lead to more consistent mappings between pitch and height, loudness and distance/height, and tempo and speed of hand movement and muscular energy. Our results corroborate previously reported pitch vs. height (higher pitch leading to higher elevation in space) and tempo vs. speed (increasing tempo leading to increasing speed of hand movement) associations, but also reveal novel findings pertaining to musical training which influenced consistency of pitch mappings, annulling a commonly observed bias for convex (i.e., rising–falling) pitch contours. Moreover, we reveal effects of interactions between musical parameters on cross-modal mappings (e.g., pitch and loudness on speed of hand movement), highlighting the importance of studying auditory stimuli concurrently varied in different musical parameters. Results are discussed in light of cross-modal cognition, with particular emphasis on studies within (embodied) music cognition. Implications for theoretical refinements and potential clinical applications are provided.

## Introduction

### Origin and shaping of cross-modal correspondences

Research on cross-modal correspondences has shown that people readily map features of auditory stimuli such as pitch and loudness onto the visual or visuo-spatial domain (for reviews see e.g., Marks, 2004; Spence, 2011; Eitan, 2013a). The most extensively studied cross-modal correspondence—that of pitch height (henceforth “pitch”) and spatial height—has produced robust effects revealing that higher (lower) pitch is associated with higher (lower) elevation in space (Pratt, 1930; Trimble, 1934; Miller et al., 1958; Pedley and Harper, 1959; Mudd, 1963; Roffler and Butler, 1968; Bernstein and Edelstein, 1971; Bregman and Steiger, 1980; Melara and O'Brien, 1987; Walker, 1987; Miller, 1991; Ben-Artzi and Marks, 1995; Patching and Quinlan, 2002; Casasanto et al., 2003; Widmann et al., 2004; Rusconi et al., 2006; Cabrera and Morimoto, 2007; Mossbridge et al., 2011). It is unclear, however, what exactly the reason for this cross-modal correspondence is. Different causes of cross-modal mappings have been proposed, e.g., macro-level factors such as development, statistical learning, or culture more generally, and micro-level factors pertaining to experimental paradigms and stimuli selection.

With regard to the impact of culture, there is evidence that the kinds of mappings adults display are influenced by language (Dolscheid et al., 2013), emphasizing the importance of conceptual metaphor (Johnson and Larson, 2003; Eitan and Timmers, 2010), which had already been identified by Carl Stumpf as the key mechanism underlying spatial mappings of pitch (Stumpf, 1883). Another cultural factor is musical training: trained individuals map auditory features more consistently than untrained individuals, but the kinds of mappings remain consistent across most Western individuals (Eitan and Granot, 2006; Küssner and Leech-Wilkinson, 2014). While culture, and particularly language, thus plays a pivotal role in *shaping* cross-modal correspondences, a growing body of research suggests that their *origin* is to be found elsewhere (but see also Deroy and Auvray, 2013). For instance, studies with infants indicate that 3–4-month-olds show pitch vs. height and pitch vs. sharpness associations (Walker et al., 2010), 4-month-olds pitch vs. height and pitch vs. thickness associations (Dolscheid et al., 2012), and 3–4-week-olds loudness vs. brightness associations (Lewkowicz and Turkewitz, 1980). Combined with evidence from audio-visual mappings in non-human mammals (Ludwig et al., 2011), this has led some scholars to conclude that cross-modal correspondences are innate, possibly based on a wide range of neural connections that are gradually lost due to synaptic pruning (Mondloch and Maurer, 2004; Wagner and Dobkins, 2011). Others have argued that cross-modal correspondences may be learned rapidly through external, non-linguistic stimulation (Ernst, 2007; as discussed in Spence, 2011) or may be acquired indirectly in cases where the occurrence of cross-modal pairings in the environment seems unlikely (Spence and Deroy, 2012). Further evidence supporting the prelinguistic origin hypothesis comes from studies showing that cross-modal mappings are processed at an early, perceptual level (Maeda et al., 2004; Evans and Treisman, 2010), unmediated by later, semantic processing (Martino and Marks, 1999; but see also Chiou and Rich, 2012). Finally, it is important to highlight the role of common neural substrates of cross-modal correspondences (Spence, 2011), which might be best accounted for by neurocomputational models.

### Complexity of audio-visuo-spatial correspondences

As implied above, cross-modal correspondences between auditory features and the visuo-spatial domain are manifold, sometimes referred to as one-to-many and many-to-one correspondences (Eitan, 2013a). For instance, pitch has been associated with vertical height (Walker, 1987), distance (Eitan and Granot, 2006), speed (Walker and Smith, 1986), size (Mondloch and Maurer, 2004) and brightness (Collier and Hubbard, 1998)—i.e., one-to-many—while the same associations have been found for loudness (Lewkowicz and Turkewitz, 1980; Neuhoff, 2001; Lipscomb and Kim, 2004; Eitan et al., 2008; Kohn and Eitan, 2009), rendering, for example, pitch/loudness vs. height a many-to-one correspondence. The full story is, however, more complex than that, as outlined in Eitan (2013a). First, the type of auditory stimuli, whether static or dynamic, can give rise to opposing results. For instance, static high and low pitches paired with small and large visual disks, respectively, have been shown to enhance performance in a speeded classification paradigm (Gallace and Spence, 2006), providing evidence that high pitch is associated with small objects and low pitch with large objects. On the other hand, Eitan et al. (2013), using a similar paradigm, demonstrated that rising pitches paired with an increasing visual object and falling pitches paired with a decreasing visual object yielded significantly faster responses than rising pitches paired with a decreasing visual object and falling pitches with an increasing visual object. Secondly, manipulating several auditory features concurrently influences participants' cross-modal images of motion (Eitan and Granot, 2011). For instance, an increase in tempo, usually associated with an increase in speed, did not lead to an increase in speed when loudness was concurrently decreasing. Similarly, a rise in pitch, usually associated with an increase in vertical position, led to a *decrease* in vertical position when loudness was concurrently decreasing. Since environmental sounds, but especially music, are very often varied *dynamically and concurrently* in pitch, loudness, tempo, timbre etc., investigating cross-modal correspondences of these features—which is frequently done by manipulating them in isolation, entailing obvious experimental advantages but also the even more obvious lack of ecological validity—requires approaches taking into consideration the multiple dynamic co-variations of sound features.

### Cross-modal mappings of sound involving real or imagined bodily movements

Whereas most experimental paradigms to date have used speeded identification, speeded classification or forced-choice matching tasks, researchers have recently begun to apply paradigms involving real-time drawings (Küssner and Leech-Wilkinson, 2014), gestures (Kozak et al., 2002) or imagined bodily movements (Eitan and Granot, 2006), in order to delineate a more differentiated picture of cross-modal mappings. Asking participants to imagine the movements of a humanoid character in response to changes in a range of musical parameters, Eitan and Granot (2006) found that pitch is mapped onto all three spatial axes, including asymmetric pitch vs. height mappings such that decreasing pitch was more strongly associated with descending movements than increasing pitch with ascending movements. Similarly, the authors report two asymmetric mappings of loudness: (1) decreasing loudness was more strongly associated with spatial descent than increasing loudness with spatial ascent, and (2) increasing loudness was more strongly associated with accelerating movements than decreasing loudness with decelerating movements. What is more, results from a study investigating participants' perceptions of the congruency between vertical arm movements and changes in pitch and loudness revealed that concurrent rising–falling movements of one's arm and pitch or loudness gave rise to higher ratings than concurrent falling–rising movements (Kohn and Eitan, 2012). These striking asymmetries might be part of a discrepancy between response-time or rating paradigms and those involving more extensive, overt bodily movements.

A few studies only have investigated how changes in auditory stimuli are mapped onto real bodily movements. In an exploratory study, Godøy et al. (2006) asked participants to respond with hand gestures—captured with a pen on an electronic graphics tablet—to a set of auditory stimuli that comprised instrumental, electronic and environmental sounds and was classified according to a typology developed by Pierre Schaeffer (e.g., impulsive, continuous and iterative sounds). While the authors report a “fair amount of consistency in some of the responses” such as ascending movements for increasing pitch, they do stress the need for large-scale studies involving the investigation of free movements in three-dimensional space as well as of the influence of musical training. In a subsequent study from the same group, Nymoen et al. (2011) found strong associations between pitch and vertical movements, between loudness and speed, and between loudness and horizontal movements, when comparing people's gestural responses to pitched and non-pitched sounds, captured by moving a rod whose movements were supposed to represent sound-producing gestures. While the authors' argument for “a one-dimensional intrinsic relationship between pitch and vertical position” is conceivable in view of their findings, the lack of bidirectional pitch changes (e.g., rising–falling contour) within their auditory stimuli precludes conclusions about potential asymmetric mappings of pitch with bodily movements.

In a similar experiment, Caramiaux et al. (2014) compared hand gestures in response to action and non-action related sounds, confirming their hypothesis that the former would entail sound-producing gestures while the latter would result in gestures representing the sound's spectromorphology (Smalley, 1997), i.e., the overall sonic shape. Comparing speed profiles between participants revealed that they were more similar for non-action- than action-related sounds. This shows—and is supported by analysis of interviews carried out with the participants—that once a particular action (e.g., crushing a metallic can) has been identified, the realization of the accompanying gesture is highly idiosyncratic. On the other hand, non-action-related sounds, which are particularly pertinent to the present study, gave rise to more consistent gestural responses.

One study has been carried out investigating free representational movements to sound, in which 5- and 8-year-old children were presented with auditory stimuli separately varied in pitch, loudness and tempo (Kohn and Eitan, 2009). Three independent referees trained in Laban Movement Analysis rated the observed behavior—the sound being muted—according to the movement and direction along the x-, y- and z-axes, the muscular energy and the speed. Pitch was most strongly associated with the vertical axis, loudness with vertical axis and muscular energy, and tempo with speed and muscular energy. In terms of direction, changes in loudness and tempo gave rise to congruent movement patterns, that is, increasing loudness was represented with upward movement and higher muscular activity, whereas decreasing loudness was represented with downward movement and lower muscular activity. The direction of movement along the vertical axis in response to changes in pitch was congruent for increasing–decreasing pitch contours but not for decreasing–increasing contours. This finding is particularly relevant for the present study, as it highlights the asymmetric nature of bodily cross-modal mappings.

### Aims and hypotheses

To sum up, there is currently a lack of studies investigating (a) how auditory stimuli concurrently varied in several sound features are mapped cross-modally and (b) how approaches involving gestural (i.e., bodily) responses affect cross-modal correspondences. To address this gap, and to provide a starting point for researchers to develop further testable hypotheses, the present exploratory study aims to identify how pitch, loudness and tempo are represented gesturally in real-time (i.e., occurring simultaneously with latencies <100 ms), and to what extent musical training influences those cross-modal mappings. Unlike studies investigating the influence of musicians' specializations on cross-modal mappings of sound—such as pianists' horizontal pitch mappings (Stewart et al., 2013; Lega et al., 2014, Exp. 2; Taylor and Witt, 2014)—we are concerned with the influence of more generic musical skills (e.g., the ability to read music notation) acquired in contexts of formal music education, and thus aim to balance our trained participants' main musical activity more carefully than previous studies (Rusconi et al., 2006; Lidji et al., 2007). To our knowledge, this is the first controlled experiment studying adults' gestural responses to a set of pure tones systematically and concurrently varied in pitch, loudness and tempo. Based on the literature reviewed above, we hypothesize the following outcomes:

1. Pitch is represented on the y-axis (higher elevation for higher pitches); rising–falling pitch contours (convex shapes) are expected to yield greater pitch vs. height associations than falling–rising pitch contours.
2. Loudness is represented with forward-backward movements along the z-axis and muscular energy (forward movement/more energy for louder sounds), as well as with spatial height when loudness is the only auditory feature being manipulated (higher elevation for louder sounds).
3. Tempo of pitch change in the auditory stimuli is represented by speed of the hand movements (faster movement for faster tempo) and muscular energy (more energy for faster tempo).
4. Musical training has an impact such that musically trained participants—due to their formalized engagement with musical parameters (e.g., through notation)—show generally more consistent mappings than musically untrained participants.

## Materials and methods

### Participants

Sixty-four participants (32 female) took part in the experiment (age: *M* = 29.63 years, *SD* = 12.49 years, range: 18–74 years). Thirty-two participants (16 female) were classified as musically trained (age: *M* = 30.09 years, *SD* = 13.66 years, range: 18–74 years), and 32 (16 female) as musically untrained (age: *M* = 29.16 years, *SD* = 11.39 years, range: 18–67 years). All participants were required to be 18 years or over, right-handed, and must not have been diagnosed with any vision or hearing impairments (except those corrected to normal vision with glasses or contact lenses). To satisfy the “musically trained” category, participants must have played either a keyboard instrument, a string instrument, a wind/brass instrument or been a composer, must have had at least Grade 8 of the ABRSM system (http://gb.abrsm.org/en/home) or an equivalent qualification, and must have spent at least 4 h per week on average playing their respective main instrument or composing. All musically trained participants were balanced by gender and main musical activity. Musically untrained participants must not have played any musical instrument or composed music for the past 6 years, must not have played any instrument for more than 2 years in total, and must not have exceeded Grade 1 ABRSM. Participants were recruited using a college-wide e-mail recruitment system including undergraduates, postgraduates and staff, as well as circulating a call for participants within music conservatoires. All criteria were clearly stated in the recruitment email and checked again with a questionnaire during the experiment. Exceptions included one trained participant who reported playing only 2 h on average per week and one untrained participant who reported engaging in musical activities (“electronics, drums, mixing”) for 7.5 h. Another musically untrained participant had played the guitar for 4 years in total but had stopped playing 14 years ago, and one untrained participant who had played drums for 1 year had only stopped 5 years ago. Since this study is concerned with differences arising from formal training, and none of the musically untrained participants had taken any formal music examination while all musically trained participants were at Grade 8 or above, it was decided to keep all participants for the analysis to ensure a balanced design and sufficient statistical power.

### Stimuli

**Figure 1:**
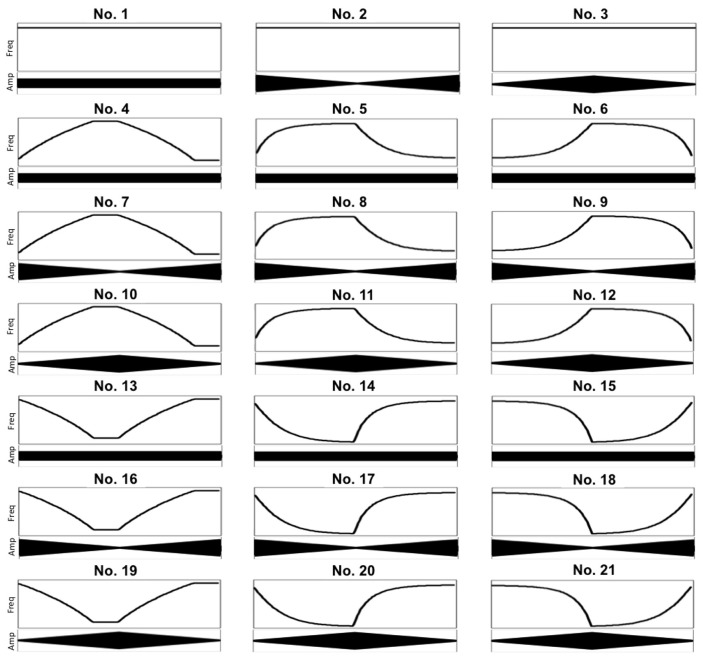
**Overview of frequency and amplitude contours of experimental sound stimuli**. All x-axes represent time (length of stimuli: 8 s). Highest/lowest frequency: 123.47/293.67 Hz. Equal amplitude means 50% of the maximum, decreasing amplitude means 90–10% of the maximum and increasing amplitude means 10–90% of the maximum. Freq, log frequency (Hz); Amp, amplitude.

Stimuli (see Table 1, Figure 1 and Supplementary Material) were synthesized in SuperCollider (Version 3.5.1) and consisted of 21 continually sounding pure tones that varied in frequency, amplitude and tempo. All stimuli were 8 s long. For a pure tone stimulus, pitch height is the subjective quality that covaries with the frequency of the tone, other acoustic parameters held constant. Trough and peak pitches were B2 (123.47 Hz) and D4 (293.67 Hz), respectively, and all but three stimuli (Nos. 1–3) had a rising–falling (Nos. 4–12) or falling–rising (Nos. 13–21) pitch contours. Constant amplitude meant 50% of the maximum, whereas stimuli linearly decreasing and increasing in amplitude showed the pattern 90% – 10% – 90% (reaching 10% after 4 s) and stimuli linearly increasing and decreasing in amplitude showed the pattern 10% – 90% – 10% (reaching 90% after 4 s). Given 100% full scale amplitude = 0 dB, 50% = −3.01 dB, 90% = −0.46 dB, and 10% = −10 dB. Stimuli changing in pitch reached the top (bottom) after 3 s, before going into the opposite direction after 1 s and reaching the bottom (top) after 3 s and staying there for another second. The factors for change in tempo were −0.5 for decelerandi and 0.5 for accelerandi. Each decelerando/accelerando lasted 4 s.

**Table 1:** **Overview of experimental sound stimuli**.

**No**.	**Frequency (note name)**	**Amplitude**	**Tempo**
1	Constant (D4)	Constant	Not applicable
2	Constant (D4)	Decreasing–Increasing	Not applicable
3	Constant (D4)	Increasing–Decreasing	Not applicable
4	Rising–Falling (B2–D4–B2)	Constant	Equal
5	Rising–Falling (B2–D4–B2)	Constant	Decelerando–Decelerando
6	Rising–Falling (B2–D4–B2)	Constant	Accelerando–Accelerando
7	Rising–Falling (B2–D4–B2)	Decreasing–Increasing	Equal
8	Rising–Falling (B2–D4–B2)	Decreasing–Increasing	Decelerando–Decelerando
9	Rising–Falling (B2–D4–B2)	Decreasing–Increasing	Accelerando–Accelerando
10	Rising–Falling (B2–D4–B2)	Increasing–Decreasing	Equal
11	Rising–Falling (B2–D4–B2)	Increasing–Decreasing	Decelerando–Decelerando
12	Rising–Falling (B2–D4–B2)	Increasing–Decreasing	Accelerando–Accelerando
13	Falling–Rising (D4–B2–D4)	Constant	Equal
14	Falling–Rising (D4–B2–D4)	Constant	Decelerando–Decelerando
15	Falling–Rising (D4–B2–D4)	Constant	Accelerando–Accelerando
16	Falling–Rising (D4–B2–D4)	Decreasing–Increasing	Equal
17	Falling–Rising (D4–B2–D4)	Decreasing–Increasing	Decelerando–Decelerando
18	Falling–Rising (D4–B2–D4)	Decreasing–Increasing	Accelerando–Accelerando
19	Falling–Rising (D4–B2–D4)	Increasing–Decreasing	Equal
20	Falling–Rising (D4–B2–D4)	Increasing–Decreasing	Decelerando–Decelerando
21	Falling–Rising (D4–B2–D4)	Increasing–Decreasing	Accelerando–Accelerando

### Motion capture

A Microsoft^®^ Kinect™ was used to capture participants' hand movements. Further technical details (e.g., spatial resolution) can be found at http://openkinect.org/wiki/Imaging_Information. The bespoke software for the purposes of this experiment was developed in Processing v1.2.1 (Fry and Reas, 2011). The whole experimental session was recorded with two video cameras (Panasonic HDC-SD 700/800). Participants also held a Nintendo^®^ Wii™ Remote Controller in the same hand that was performing the gestures. If the latter was shaken strongly enough for the acceleration threshold of 10 m/s^2^ to be exceeded, the fast shaking hand movements were recorded by the software (see Section Data Analysis).

### Procedure

After signing the consent form, participants read detailed instructions and any remaining uncertainties were discussed with the experimenter. Participants were introduced to the Kinect™ and Wii™ Remote Controller technologies, made aware of the experimental space, and familiarized with the noise-canceling headphones to be worn during the experiment (Bose QuietComfort^®^ 15 Acoustic Noise Canceling^®^). The volume of the stimuli was set at a comfortable level by the experimenter and kept constant for all participants. The participants' task was to represent the sound stimuli with their right hand in which they held the Wii™ Remote Controller; it was stressed that (a) there were no “right” or “wrong” responses, (b) participants' responses should be consistent such that, if the same sound occurred twice, they should make the same movement, and (c) they should try to represent gesturally all sound characteristics they are able to identify.

The whole experiment consisted of four parts, including musical excerpts and a real-time visualization on a screen in front of the participants (Küssner, 2014). Here, we will only report the results of the first part, in which participants gestured in response to pure tones without seeing a visualization in front of them. After a short calibration procedure with the Kinect™ to identify and track the participants' right hand, a summary of the instructions appeared on the screen. Once participants were ready, they informed the experimenter, who was seated behind another screen and was not able to see their movements, and the first block—practice trials consisting of five pure tones (see Supplementary Material)—was started. If participants did not have any further questions after the practice trials (they could repeat the practice trials as often as they wished), the second block consisting of all 21 pure tones was started. The presentation order of stimuli within the blocks was randomized. Participants were presented with each stimulus twice consecutively. The first time, they were supposed to listen only: 2 s prior to the stimulus onset the instruction “Get ready to LISTEN. X stimuli left. [countdown]” appeared in the upper left corner of the screen, informing participants about the number of stimuli left in this block (X) and starting a short countdown. The second time, participants were supposed to represent the sound stimulus gesturally while it was played. The instruction “Get ready to GESTURE. [countdown]” appeared and participants were again prepared for the onset of the stimulus with a countdown. This procedure had been approved by the College Research Ethics Committee (REP-H/10/11-13).

### Data analysis

The sound features—frequency in Hz and estimated loudness in sone, sampled at 20 Hz each—were extracted with Praat version 5.3.15 (Boersma and Weenink, 2012). Frequency values were log-transformed to account for human perception of pitch, as is common practice in psychophysical experiments (e.g., see Micheyl et al., 2006). Both log-transformed frequency values and loudness values were then standardized (*M* = 0, *SD* = 1) per sound stimulus. The Kinect™ data—X, Y and Z coordinates sampled at ca. 15 Hz [mean frame length was 66.24 ms (*SD* = 4.95 ms) and median 68 ms]—were extracted together with their timestamps. All three spatial coordinates were then standardized (*M* = 0, *SD* = 1) per sound stimulus. Next, sound features were linearly interpolated to realign them with the movement data at the timestamps of the Kinect™ data, creating a matrix with six columns (timestamp, frequency, loudness, X, Y, Z) per stimulus.

As an indicator of the degree of the association between sound and movement features Spearman's rho—a non-parametric correlation coefficient—was calculated. This measure has been suggested for time-dependent data by Schubert (2002), and has been used by various scholars for similar datasets (e.g., Vines et al., 2006; Nymoen et al., 2013; Küssner and Leech-Wilkinson, 2014). It has been argued that one needs to be cautious when interpreting the size of correlation coefficients derived from time-dependent data. For Spearman's rho, this is even more straightforward: regardless of time-dependence, the absolute size of this coefficient is never interpretable because its variance is not defined. Though the significance of a single Spearman's rho correlation coefficient derived from a time-dependent dataset might not be meaningful, it can be valuable to compare several correlation coefficients.

For the purpose of this analysis, global and local Spearman's correlation coefficients were computed. The number of data points for a local correlation was *N* = 119, and for a global correlation *N* = 2142. Only sound stimuli Nos. 4–21 were entered into the analysis (unless stated otherwise) since stimuli Nos. 1–3 contain constant features which cannot be entered into a correlation analysis. Note that the loudness was only genuinely equal in stimulus No. 1. Due to the equal-loudness-level contours for pure tones (Suzuki and Takeshima, 2004) and the use of loudness measured in sone, stimuli Nos. 4–6 and 13–15, whose amplitude was constant, could be entered into the analysis because their perceived loudness varied marginally according to the pitch contour.

“Global” denotes the correlation between sound features of all stimuli of a single participant and their accompanying hand movements (e.g., global frequency–Y correlation coefficient of participant *k*). “Local” denotes the correlation between sound features of a particular stimulus of a single participant and their accompanying hand movements (e.g., local frequency–Y correlation coefficient of sound stimulus *s* of participant *k*).

The following analytical steps were applied to investigate gestural representations of pitch and loudness and carried out in IBM SPSS Statistics (Version 20). First, the absolute global correlation coefficients between frequency and loudness and, respectively, the three spatial axes X, Y and Z, were entered into two ANOVAs with the within-subjects factor “space” (X/Y/Z) to identify the three strongest correlations between each variable and each of the three axes (e.g., frequency and Y), which was then examined further in the subsequent steps of the analysis. Secondly, the original (rather than absolute) global correlation coefficients were examined to identify the direction of movement. Thirdly, the effects of interactions between musical parameters (pitch contour, loudness contour and tempo) on the size of the correlations were investigated by means of local correlation coefficients, resulting in ANOVAs with the between-subjects factor “training” (musically trained/musically untrained) and the within-subjects factors “pitch” (rising–falling/falling–rising), “loudness” (constant amplitude/decreasing–increasing/increasing–decreasing) and “tempo” (equal/decelerando–decelerando/accelerando–accelerando). All *post-hoc* pairwise comparisons were Sidak-corrected. Fourthly, to investigate whether muscular energy of the hand was associated with loudness and tempo variations in the stimuli, data from the Wii™ Remote Controller were collected when the difference in acceleration between the current and previous frame exceeded 10 m/s^2^. That is, when participants shook the Controller (henceforth “shaking event”) strongly enough, the software recorded a shaking event with a timestamp. Fifthly, to investigate whether the speed of the hand movement was associated with tempo variations in the stimuli, the mean velocity in response to each quarter of a sound stimulus was the dependent variable of an ANOVA with the within-subjects factors “half” (1st/2nd half of a stimulus), “quarter” (1st/2nd quarter of each half), “pitch” (up/down), “loudness” (constant amplitude/decreasing/increasing) and “tempo” (equal/decelerando/accelerando), and the between-subjects factor “training.” Whenever the assumption of sphericity was violated in repeated-measures ANOVAs, the degrees of freedom were adjusted using the Greenhouse-Geisser correction. Any follow-up *t*-tests were Bonferroni-corrected.

## Results

### Pitch

#### Absolute global correlation analysis

There was a main effect of “space” [*F*_(1.75,110.47)_ = 192.87, *p* < 0.001, partial η^2^ = 0.75], and all three Sidak-corrected pairwise comparisons revealed significant differences (all *p* < 0.001). Correlations with Y (*M* = 0.68, s.e.m. = 0.03) were greater than with X (*M* = 0.14, s.e.m. = 0.02) and with Z (*M* = 0.25, s.e.m. = 0.02), and correlations with Z were greater than with X. All 64 participants showed positive correlation coefficients, suggesting that they moved their hand upwards with increasing pitch and downwards with decreasing pitch. We thus shift our focus to the analysis of local correlations of frequency–Y.

#### Interactions between musical parameters—local correlations of frequency–Y

**Figure 2:**
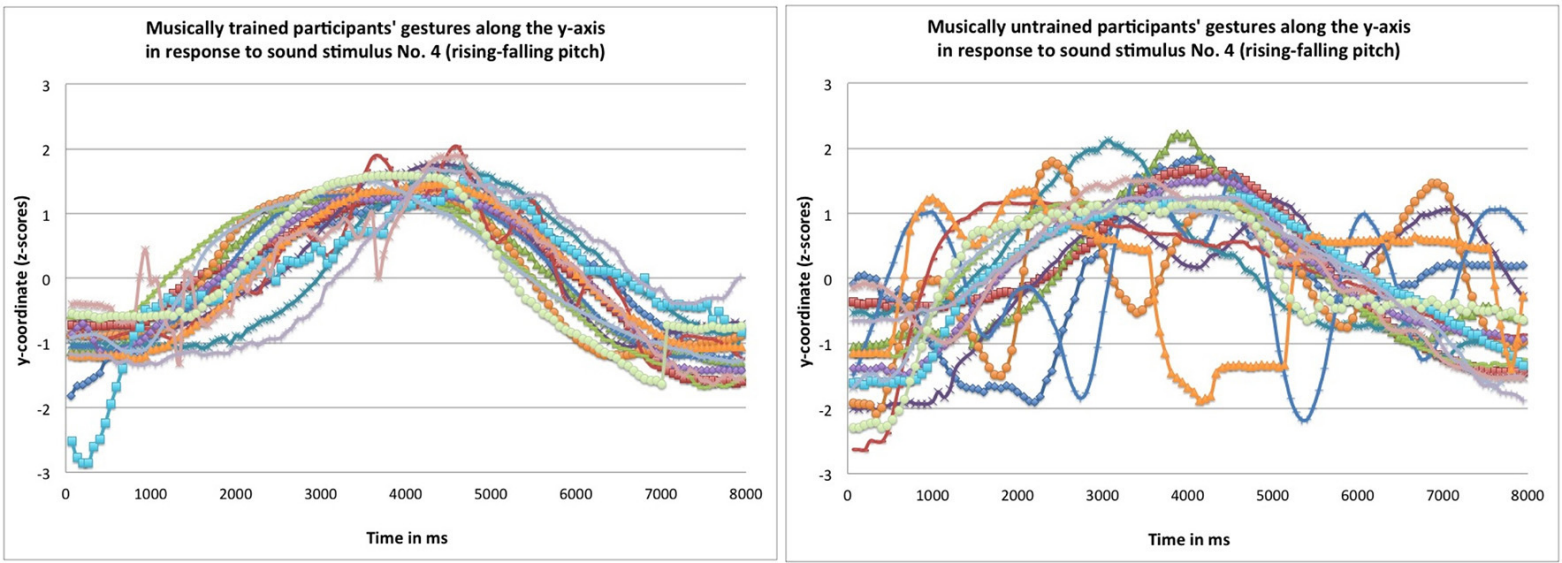
**Gestural trajectories along the y-axis in response to sound stimulus rising and falling in pitch (No. 4) by a subsample of 16 randomly chosen musically trained participants (left) and 16 randomly chosen musically untrained participants (right)**.

Results revealed main effects of “training” [*F*_(1, 62)_ = 18.64, *p* < 0.001, partial η^2^ = 0.23], “pitch” [*F*_(1, 62)_ = 5.04, *p* = 0.028, partial η^2^ = 0.08], “loudness” [*F*_(2, 124)_ = 3.44, *p* = 0.035, partial η^2^ = 0.05], and “tempo” [*F*_(1.55,96.34)_ = 8.29, *p* = 0.001, partial η^2^ = 0.12]. The positive association between pitch and height was larger for musically trained (*M* = 0.79, s.e.m. = 0.03) compared to untrained participants (*M* = 0.58, s.e.m. = 0.03); rising–falling pitch contours (*M* = 0.71, s.e.m. = 0.02) led to higher frequency-Y correlation coefficients than falling–rising pitch contours (*M* = 0.65, s.e.m. = 0.03); constant amplitude (*M* = 0.71, s.e.m. = 0.02) gave rise to higher frequency-Y correlation coefficients than decreasing–increasing loudness contours (*M* = 0.67, s.e.m. = 0.03, *p* = 0.042); and equal tempo (*M* = 0.73, s.e.m. = 0.03) compared to both “accelerando–accelerando” (*M* = 0.64, s.e.m. = 0.03, *p* = 0.004) and “decelerando–decelerando” (*M* = 0.68, s.e.m. = 0.03, *p* = 0.018) resulted in higher frequency–Y correlation coefficients. Primary response data—gestural trajectories along the y-axis in response to sound stimulus No. 4 (rising–falling pitch)—are shown for a subsample of 16 randomly chosen musically trained participants (Figure 2 left) and 16 randomly chosen musically untrained participants (Figure 2 right).

**Figure 3:**
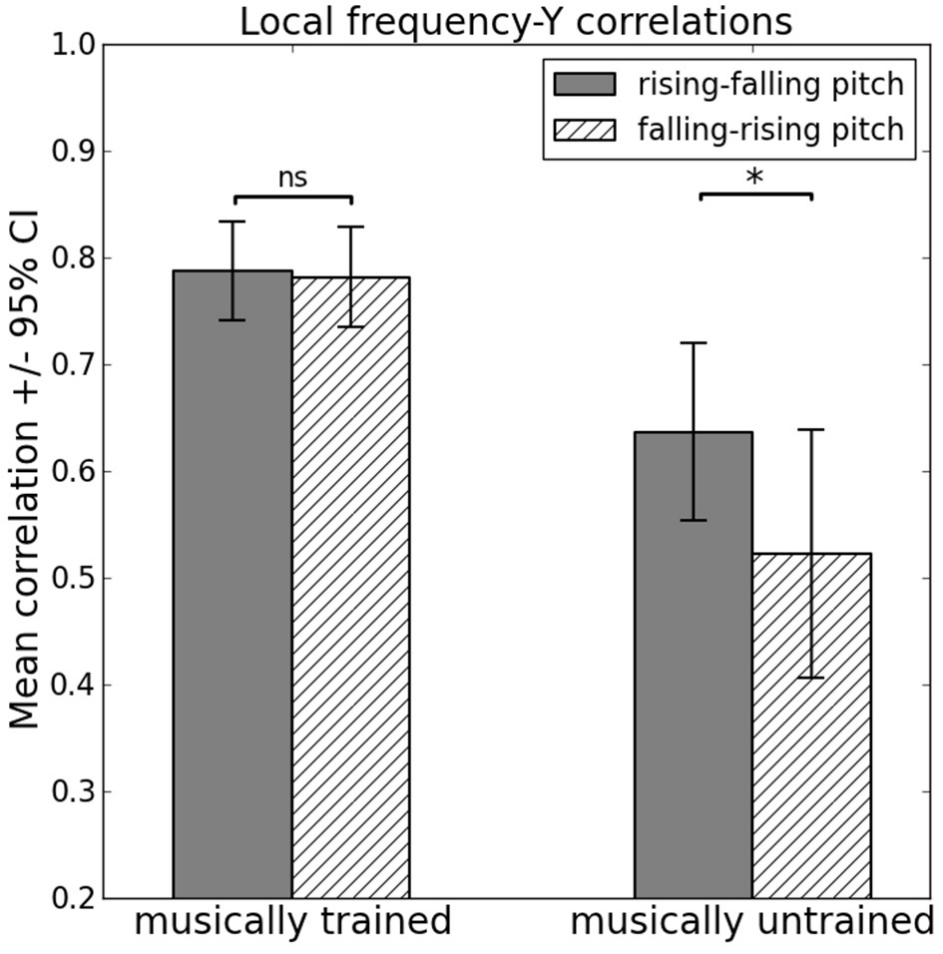
**Influence of interaction between musical training and pitch contour on local frequency–Y correlations**. ^*^ indicates *p* < 0.05, ns, not significant.

Several two- and three-way interactions were observed. There was a significant interaction effect between “pitch” and “training” [*F*_(1, 62)_ = 4.12, *p* = 0.047, partial η^2^ = 0.06], revealing that the observed main effect of “pitch” is chiefly due to musically untrained participants' lower frequency–Y correlation coefficients when presented with falling–rising pitch contours (*M* = 0.52, s.e.m. = 0.04) compared to rising–falling pitch contours (*M* = 0.64, s.e.m. = 0.03), *t*_(31)_ = 2.32, *p* = 0.027, *r* = 0.38. In comparison, musically trained participants' frequency–Y correlation coefficients did not differ significantly [rising–falling pitch contours: *M* = 0.79, s.e.m. = 0.03; falling–rising pitch contours: *M* = 0.78, s.e.m. = 0.04; *t*_(31)_ = 0.28, *p* = 0.781, *r* = 0.05], see Figure 3.

There were also significant interaction effects between “tempo” and “training” [*F*_(2, 124)_ = 10.10, *p* < 0.001, partial η^2^ = 0.14], between “pitch” and “tempo” [*F*_(1.46,90.44)_ = 20.00, *p* < 0.001, partial η^2^ = 0.24], between “pitch” and “loudness” [*F*_(1.73,107.24)_ = 5.30, *p* = 0.009, partial η^2^ = 0.08], between “pitch,” “tempo” and “loudness” [*F*_(4, 248)_ = 3.85, *p* = 0.005, partial η^2^ = 0.06], and between “pitch,” “tempo” and “training” [*F*_(1.46,90.44)_ = 4.45, *p* = 0.024, partial η^2^ = 0.07]. Running nine follow-up *t*-tests (alpha level: 0.0056) to compare frequency–Y correlation coefficients of rising–falling and falling–rising pitch contours across different loudness and tempo profiles, two significant effects were found. Together with equal amplitude and “accelerando–accelerando” pattern, the rising–falling pitch contour (*M* = 0.76, s.e.m. = 0.04) led to higher frequency–Y correlation coefficients than the falling–rising pitch contour [*M* = 0.59, s.e.m. = 0.05; *t*_(63)_ = 3.05, *p* = 0.003, *r* = 0.36]. Similarly, together with increasing–decreasing amplitude and “accelerando–accelerando” pattern, the rising–falling pitch contour (*M* = 0.81, s.e.m. = 0.03) gave rise to higher frequency–Y correlation coefficients than the falling–rising pitch contour [*M* = 0.46, s.e.m. = 0.06; *t*_(63)_ = 6.21, *p* < 0.001, *r* = 0.62]. Although not significant [*t*_(63)_ = 2.75, *p* = 0.008, *r* = 0.33], the same trend was observed for stimuli with decreasing–increasing amplitude and “accelerando–accelerando” pattern.

Further support for the observation that “accelerando–accelerando” patterns increase the difference of frequency–Y correlation coefficients between rising–falling and falling–rising pitch contours is provided by breaking down the interaction between “pitch,” “tempo” and “training.” It was revealed that both musically trained [*t*_(31)_ = 2.82, *p* = 0.008, *r* = 0.45] and musically untrained participants [*t*_(31)_ = 5.10, *p* < 0.001, *r* = 0.68] showed higher frequency–Y correlation coefficients when “accelerando–accelerando” patterns were paired with rising–falling (trained: *M* = 0.84, s.e.m. = 0.02; untrained: *M* = 0.66, s.e.m. = 0.05) compared to falling–rising pitch contours (trained: *M* = 0.75, s.e.m. = 0.04; untrained: *M* = 0.31, s.e.m. = 0.06).

### Loudness

#### Absolute global correlation analysis

Results revealed a main effect of “space” [*F*_(2, 126)_ = 108.49, *p* < 0.001, partial η^2^ = 0.63]. Whereas correlations with Y (*M* = 0.33, s.e.m. = 0.01) were greater than with X (*M* = 0.10, s.e.m. = 0.01) and with Z (*M* = 0.14, s.e.m. = 0.01; both *p* < 0.001), correlations with Z did not differ from correlations with X (*p* = 0.170). Apart from one musically untrained participant (ρ = −0.001), all participants showed positive correlations between loudness and height, suggesting that they moved their arm upwards with increasing loudness and downwards with decreasing loudness. The question arises, however, whether participants indeed chose to represent loudness with the y-axis, or whether this is a spurious effect, caused by interactions between pitch and loudness in the stimuli. Recall that stimuli Nos. 10–12 and 16–18 consist, respectively, of concurrently increasing–decreasing and decreasing–increasing pitch and loudness contours, whereas stimuli Nos. 7–9 and 19–21 consist of opposing pitch and loudness contours (see Figure 1). Thus, it is vital to consider the local correlations to identify whether the positive loudness–Y correlations values are in fact a side effect of frequency–Y correlation coefficients. If so, there should be a significant interaction effect between “pitch” and “loudness,” resulting in negative loudness–Y correlations for stimuli when the pitch contour is rising–falling (falling–rising) and the loudness contour is concurrently decreasing–increasing (increasing–decreasing).

#### Interactions between musical parameters—local correlations of loudness–Y

**Figure 4:**
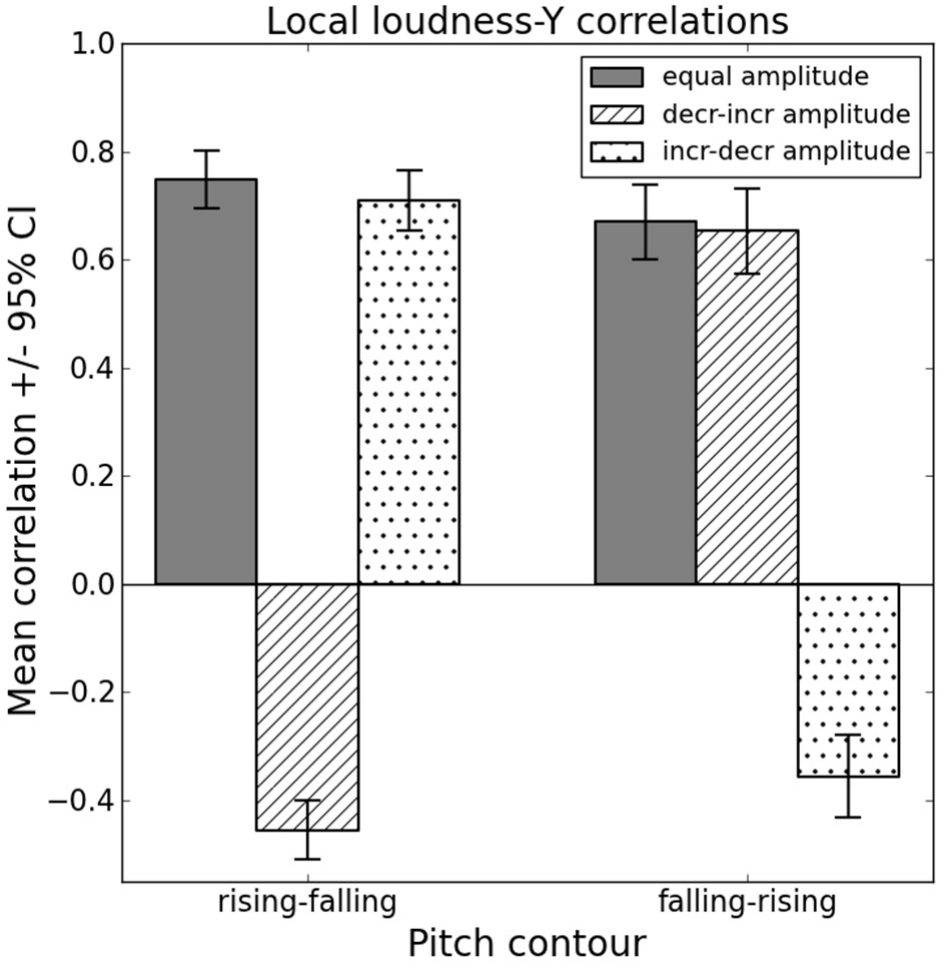
**Spurious loudness vs. height association: influence of interaction between pitch and loudness contour on local loudness–Y correlations**.

Although there are main effects of “training,” “loudness” and “tempo,” as well as two-way interactions between “loudness” and “training,” “tempo” and “training,” “pitch” and “tempo,” and “loudness” and “tempo,” the main focus here is on a highly significant interaction between “pitch” and “loudness” [*F*_(1.46,90.60)_ = 481.88, *p* < 0.001, partial η^2^ = 0.89]. Inspecting the interaction graph (see Figure 4), it becomes obvious that participants map pitch, not loudness, onto the y-axis. When rising–falling pitch contours are paired with decreasing–increasing loudness contours the loudness–Y correlation coefficients are negative (*M* = −0.45, s.e.m. = 0.03), and when paired with increasing–decreasing loudness contours they are positive (*M* = 0.71, s.e.m. = 0.03). Similarly, when falling–rising pitch contours are paired with increasing–decreasing loudness contours the loudness–Y correlation coefficients are negative (*M* = −0.36, s.e.m. = 0.04), and when paired with decreasing–increasing loudness contours they are positive (*M* = 0.65, s.e.m. = 0.04). Also the slight decrease of loudness–Y correlation coefficients from rising–falling (*M* = 0.75, s.e.m. = 0.03) to falling–rising pitch contours (*M* = 0.67, s.e.m. = 0.03) when the amplitude is equal fits into the picture, as it reflects the main effect of “pitch” for frequency–Y correlation coefficients.

Due to the clear results obtained from the interaction between “pitch” and “loudness,” the focus is shifted to stimuli without change in pitch to investigate whether there exist associations between loudness and height when loudness is the only auditory feature being manipulated.

Running a repeated-measures ANOVA on loudness–Y correlation coefficients of stimuli Nos. 2 and 3 with the within-subjects factor “loudness” and the between-subjects factor “training,” a main effect of “loudness” was observed, *F*_(1, 62)_ = 4.86, *p* = 0.031, partial η^2^ = 0.07. The increasing–decreasing loudness contour (*M* = 0.36, s.e.m. = 0.05) gave rise to higher loudness–Y correlation coefficients compared to the decreasing–increasing loudness contour (*M* = 0.17, s.e.m. = 0.07).

#### Local correlations of loudness–Z for stimuli without change in pitch

Since the association between loudness and the z-axis for stimuli concurrently varied in pitch, loudness and tempo was too small to be interpreted meaningfully (mean absolute ρ = 0.14; see Section Absolute global correlation analysis), the focus is shifted to stimuli without change in pitch to investigate whether there was any association between loudness and distance from the body when loudness was the only auditory feature being manipulated. Results revealed main effects of “training” [*F*_(1, 62)_ = 6.86, *p* = 0.011, partial η^2^ = 0.10] and “loudness” [*F*_(1, 62)_ = 23.65, *p* < 0.001, partial η^2^ = 0.28]. Loudness–Z correlation coefficients were significantly larger for musically trained (*M* = 0.33, s.e.m. = 0.07) compared to untrained participants (*M* = 0.09, s.e.m. = 0.07), and significantly larger when the loudness was increasing–decreasing (*M* = 0.43, s.e.m. = 0.06) compared to decreasing–increasing (*M* = −0.01, s.e.m. = 0.07). This suggests that only musically trained participants associated loudness with the z-axis, and only if the loudness contour was increasing–decreasing.

#### Association between muscular energy (shaking events) and loudness

The question arises whether participants did represent loudness at all when musical parameters were varied concurrently, since we have just shown that participants used neither height (representation of pitch takes precedence) nor distance (correlation coefficients too low). According to our hypotheses we expected an association between loudness and muscular energy (operationalized as shaking hand movements). An ANOVA was run to investigate whether the number of shaking events significantly changed with increasing or decreasing loudness. Results revealed a main effect of “loudness” [*F*_(1, 62)_ = 11.02, *p* = 0.002, partial η^2^ = 0.15], indicating that the number of shaking events increased when the loudness was increasing (*M* = 30.83, s.e.m. = 9.24) and decreased when the loudness was decreasing (*M* = 16.72, s.e.m. = 5.57). The large variation in the data suggests that there were substantial inter-individual differences present.

### How pitch, loudness, tempo and interactions thereof influence the speed of hand movement when representing sound gesturally

Muscular energy was also hypothesized to be associated with tempo. Results of an ANOVA investigating whether the number of shaking events significantly changed with increasing or decreasing tempo were non-significant [*F*_(1, 62)_ = 1.53, *p* = 0.220, partial η^2^ = 0.02]. Thus, our hypothesis pertaining to muscular energy and tempo is rejected and the focus is shifted to speed of hand movement.

Only interaction effects of the ANOVA involving at least the factors “quarter” and either “pitch,” “loudness” or “tempo” will be reported here since the aim is to analyse how changes of speed across either half of a sound stimulus are affected by changes in pitch, loudness and tempo. There was an interaction between “quarter” and “tempo” [*F*_(2, 124)_ = 51.10, *p* < 0.001, partial η^2^ = 0.45], indicating that when tempo was equal or decreasing across two quarters, speed of hand movement was decreasing [equal: *t*_(63)_ = 5.73, *p* < 0.001, *r* = 0.59; decelerando: *t*_(63)_ = 9.17, *p* < 0.001, *r* = 0.76]. However, when tempo increased across two quarters, there was no significant change in speed of hand movement [*t*_(63)_ = −1.56, *p* = 0.123, *r* = 0.19].

Three- and four-way interaction effects further qualified the interaction between “quarter” and “tempo.” There was a significant interaction between “quarter,” “tempo” and “training” [*F*_(2, 124)_ = 8.58, *p* < 0.001, partial η^2^ = 0.12], revealing that the non-significant result for the accelerando pattern is due to musically untrained participants' lack of increase in speed. While musically trained participants' increase in speed across two quarters is significant when tempo is accelerating [*t*_(31)_ = −2.54, *p* = 0.016, *r* = 0.42], there is no difference for untrained participants [*t*_(31)_ = 0.68, *p* = 0.50, *r* = 0.12], as shown in Table 2.

**Table 2:** **Mean speed of hand movement for the interaction quarter × tempo × training**.

**Musical training**	**Tempo**					
	**Equal**		**Decelerando**		**Accelerando**	
	**1st**	**2nd**	**1st**	**2nd**	**1st**	**2nd**
Trained	0.28 (0.02)	0.23 (0.01)	0.30 (0.02)	0.21 (0.01)	0.23 (0.02)	0.25 (0.02)
Untrained	0.27 (0.03)	0.24 (0.03)	0.29 (0.03)	0.24 (0.03)	0.24 (0.02)	0.24 (0.02)

*Values in brackets are standard errors of the mean. 1st and 2nd refer to the averaged speed of quarters 1 and 3 and quarters 2 and 4, respectively*.

There was also an interaction between “half,” “quarter,” “pitch” and “tempo” [*F*_(2, 124)_ = 3.16, *p* = 0.046, partial η^2^ = 0.05] which was broken down by running one ANOVA for each half. Whereas the first half revealed no significant interaction [*F*_(2, 124)_ = 1.40, *p* = 0.252], the second half showed a significant interaction between “quarter,” “pitch” and “tempo” [*F*_(2, 124)_ = 3.54, *p* = 0.032, partial η^2^ = 0.05]. Comparing the speed of hand movement across the second half with follow-up *t*-tests (alpha level: 0.0083), it was revealed that (a) stimuli with equal tempo led to decrease in speed when pitch was rising [*t*_(63)_ = 3.46, *p* = 0.001, *r* = 0.40], but not when pitch was falling [*t*_(63)_ = 2.03, *p* = 0.046, *r* = 0.25], (b) stimuli with decreasing tempo led to decrease in speed when pitch was falling [*t*_(63)_ = 6.44, *p* < 0.001, *r* = 0.63] and when pitch was rising [*t*_(63)_ = 5.46, *p* < 0.001, *r* = 0.57], and (c) stimuli with increasing tempo only led to increase in speed when pitch was rising [*t*_(63)_ = −3.20, *p* = 0.002, *r* = 0.37], but not when pitch was falling [*t*_(63)_ = −0.57, *p* = 0.572, *r* = 0.07], as shown in Table 3.

**Table 3:** **Mean speed of hand movement for the interaction quarter × pitch × tempo (second half of stimuli)**.

**Pitch**	**Tempo**					
	**Equal**		**Decelerando**		**Accelerando**	
	**1st**	**2nd**	**1st**	**2nd**	**1st**	**2nd**
Rising	0.29 (0.02)	0.24 (0.02)	0.30 (0.02)	0.22 (0.02)	0.24 (0.01)	0.26 (0.02)
Falling	0.26 (0.02)	0.24 (0.01)	0.29 (0.02)	0.21 (0.01)	0.24 (0.02)	0.24 (0.01)

*Values in brackets are standard errors of the mean. 1st and 2nd refer to the averaged speed of quarters 3 and 4, respectively*.

There was a significant interaction between “half,” “quarter” and “loudness” [*F*_(1.74,107.96)_ = 7.73, *p* = 0.001, partial η^2^ = 0.11]. For the first half, there was a significant interaction between “quarter” and “loudness” [*F*_(2, 124)_ = 6.68, *p* = 0.002, partial η^2^ = 0.10], revealing that the speed of hand movement across the first half decreased when the amplitude was equal [*t*_(63)_ = 4.32, *p* < 0.001, *r* = 0.48] and when the amplitude was decreasing [*t*_(63)_ = 7.01, *p* < 0.001, *r* = 0.66]. No change in speed was observed when the amplitude was increasing [*t*_(63)_ = 2.34, *p* = 0.023, *r* = 0.28]. For the second half, there was also a significant interaction between “quarter” and “loudness” [*F*_(1.56,96.62)_ = 4.01, *p* = 0.030, partial η^2^ = 0.06], confirming the pattern found in the first half: the speed of hand movement across the second half decreased when the amplitude was equal [*t*_(63)_ = 5.50, *p* < 0.001, *r* = 0.57] and when the amplitude was decreasing [*t*_(63)_ = 4.86, *p* < 0.001, *r* = 0.52). No change in speed was observed when the amplitude was increasing (*t*(63) = 2.03, *p* = 0.046, *r* = 0.25]. An overview can be seen in Table 4.

**Table 4:** **Mean speed of hand movement for the interaction half × quarter × loudness**.

**Half**	**Amplitude**					
	**Equal**		**Decreasing**		**Increasing**	
	**1st**	**2nd**	**1st**	**2nd**	**1st**	**2nd**
First	0.27 (0.02)	0.23 (0.01)	0.28 (0.02)	0.22 (0.02)	0.27 (0.01)	0.24 (0.02)
Second	0.26 (0.01)	0.23 (0.01)	0.28 (0.02)	0.23 (0.01)	0.27 (0.02)	0.25 (0.02)

*Values in brackets are standard errors of the mean. 1st and 2nd refer to the averaged speed of quarters 1 and 2 (1st half) and quarters 3 and 4 (2nd half), respectively*.

## Discussion

### Summary of main findings

Asking 64 participants to represent gesturally a set of pure tones, we analyzed their representations of pitch, loudness and tempo, taking into account interactions between musical parameters within the sound stimuli. Pitch was most strongly associated with the y-axis and loudness with the y-axis as well, though the latter finding turned out to be a spurious effect caused by concurrent changes of pitch and loudness. All participants showed positive correlation coefficients between pitch and height, and this association was larger for musically trained compared to untrained participants. Rising–falling pitch contours led to higher correlation coefficients than falling–rising pitch contours, which is mainly due to musically untrained participants' lower values when presented with the latter contour. This gap was increased, equally for trained and untrained participants, when the concurrent tempo pattern consisted of accelerandi, and regardless of the accompanying loudness patterns.

Notwithstanding the spurious loudness vs. height association for stimuli concurrently varied in pitch, loudness and tempo, those stimuli that only varied in loudness did reveal loudness vs. height associations: they were larger for increasing–decreasing compared to decreasing–increasing loudness contours, and musically trained participants showed higher values than untrained participants. The hypothesized association between loudness and z-axis was only found in stimuli that only varied in loudness, and only for musically trained participants when the loudness contour was increasing–decreasing. Muscular energy was found to be increasing (decreasing) when the loudness was increasing (decreasing), but showed no association with tempo.

Finally, speed of hand movement was associated with tempo and influenced by musical training (untrained participants did not increase speed of hand movement when tempo increased) and interactions with pitch (falling pitch prevented increase in speed when tempo increased) and loudness (increasing loudness prevented change in speed of hand movement).

### Pitch

The strong association between pitch and height corroborates findings from previous studies applying a range of different paradigms such as motion imagery (Eitan and Granot, 2006), drawings (Küssner and Leech-Wilkinson, 2014), gestures (Nymoen et al., 2013) and forced choices (Walker, 1987). Musically trained participants showing higher correlation coefficients than untrained participants is in line with previous studies, too (Walker, 1987; Küssner and Leech-Wilkinson, 2014), as is the finding that rising–falling pitch contours gave rise to higher correlation coefficients than falling–rising pitch contours (Kohn and Eitan, 2012). However, we were able to show that the latter effect is heavily influenced by training, revealing that only untrained participants, but not trained participants, show more consistent associations for rising–falling pitch contours compared to falling–rising contours. What is more, this interaction was further mediated by the tempo pattern: Both musically trained and untrained participants showed higher values when pitch and tempo patterns were concurrently increasing in the first half of the stimuli (i.e., rising pitch and increase in tempo) and moving contrarily in the second half of the stimuli (i.e., falling pitch and increase in tempo) compared to when pitch and tempo patterns were moving contrarily in the first half of the stimuli (i.e., falling pitch and increase in tempo) and concurrently increasing in the second half of the stimuli (i.e., rising pitch and increase in tempo). There are at least three different factors interacting here. First, the gestural pitch vs. height representation of decreasing pitch paired with an increase in tempo is facilitated by the laws of gravity: an object falling toward the ground accelerates. Secondly, faster processing of congruent semantic correspondences such as increasing pitch and increasing tempo, which both represent increasing intensity, facilitates accelerated upward movements. The third factor needs more explanation. The type of the pitch contour (rising–falling vs. falling–rising) is evidently crucial for the resulting association between pitch and height. While the roles of natural laws and conceptual metaphors have been discussed before in the context of cross-modal mappings (Johnson and Larson, 2003), the role of the pitch contour for embodied cross-modal mappings awaits further research. One mundane explanation could be the (lack of) effort to move the hand in a higher start position: it is simply more comfortable to wait for the beginning of a trial with the arm hanging loosely beside the body.

### Loudness

The disclosure of the spurious loudness vs. height association in stimuli varied in several auditory features is perhaps not surprising for a musical culture largely based on pitch. When confronted with opposing pitch and loudness contours, participants chose to represent pitch, not loudness, on the y-axis. Importantly, this shows that pitch vs. height associations dominate loudness vs. height associations in a context of concurrently varied sound features, putting the results reported by Kohn and Eitan (2009)—that loudness vs. height associations of sound features varied in isolation are stronger than pitch vs. height associations—and the conclusion drawn by Eitan (2013a)—that the “hierarchy of musical parameters delineating musical space and motion may conflict with the parametric hierarchy assumed by many music theorists” (i.e., pitch and duration first, loudness secondary)—into perspective. Of course, this does not mean people do not display loudness vs. height mappings (Eitan et al., 2008). As shown for stimuli only varied in loudness (Nos. 2 and 3), there exists an association between loudness and the vertical axis, which is larger for increasing–decreasing than decreasing–increasing contours (see also Kohn and Eitan, 2012) and larger for musically trained compared to untrained participants. But compared to other mappings such as pitch vs. height, this association turned out to be rather weak.

Similarly, the hypothesized association between loudness and the z-axis—relating to the distance of an object (Eitan and Granot, 2006)—was almost non-existent for stimuli concurrently varied in pitch, loudness and tempo. One crucial difference between our experimental paradigm and that of Eitan and Granot—apart from the distinction between real and imagined movement—is possibly the fact that movement in Eitan and Granot's study involved the movement of an imagined humanoid character *in relation* to the stable position of the participant, whereas in the present study only one (real) person was involved. Even more importantly, moving forwards could be achieved either by moving only the arm or the whole body forwards. Thus, in both cases, though particularly in the latter, real sense of distance was unlikely to be involved.

Nevertheless, the analysis of stimuli without changes in pitch (Nos. 2 and 3) revealed a very clear pattern: increasing–decreasing loudness contours—but not decreasing–increasing loudness contours—are represented by movements along the z-axis such that an increase (decrease) in loudness led participants to move forward (backward). And, as observed several times before, musically trained participants showed higher scores than untrained participants, whose mean correlation coefficient in fact suggests a complete absence of associations between loudness and the z-axis.

The analysis of muscular energy revealed that participants' number of shaking events increased when the loudness increased and decreased when the loudness decreased. This finding is in line with previous studies investigating children's movements in response to sound stimuli (Kohn and Eitan, 2009) and adult participants who used pressure on a pen in a drawing experiment to represent loudness (Küssner and Leech-Wilkinson, 2014). Further support for the notion that loudness is associated with human movement comes from Todd et al. (2000) who report that a loud bass drum might affect the vestibular system and hence a person's sense of motion, and from Van Dyck et al. (2013) who showed that people modify their body movements according to the level of the bass drum when moving to contemporary dance music. Note that muscular energy—conceptualized in the present study as very fast (shaking) hand movements—does not account for instances in which muscles might be tense without any hand movements involved. Thus, in future studies, electromyography might be used to encompass further instances in which muscular energy is involved.

### Speed of hand movement

Although pitch had been associated with speed in adjective matching (Walker and Smith, 1986) and rating tasks before (Eitan and Timmers, 2010), no such association was found in the present study. Similarly, there was no clear association between loudness and speed—a result that might have been biased by the stimuli involved in this analysis. One third of them—i.e., the ones with equal tempo (Nos. 4, 7, 10, 13, 16, 19)—included 1 s of unchanged pitch at the end of each half of a stimulus. Previous research has indicated that musically trained participants continue drawing a horizontal line when presented with pitch unchanged over time, while untrained participants stop drawing for a moment and only continue when pitch changes again (Küssner and Leech-Wilkinson, 2014). The absence of this effect in the interaction between “quarter,” “tempo” and “training” suggests, however, that gesturing sounds produces different results from drawing sounds. It is possible that participants stopped gesturing briefly when reaching these points, creating a “slowing down” bias at the end of each half. It is most likely for the same reason that the speed of hand movement decreases across two quarters of a stimulus when the tempo is equal (see Table 2). This potential bias notwithstanding, the fact that the speed of hand movement decreased when the loudness decreased and that the speed did not change when the loudness increased suggests that loudness did have an influence. At least partly, then, this finding suggests a gap between imagined and real bodily cross-modal mappings. While Eitan and Granot (2006) found no association between decreasing loudness and decreasing speed in a rating task, the present study, as well as that of Kohn and Eitan (2009), provides evidence for such a correspondence.

The association between tempo and speed of hand movement is more straightforward. With increasing tempo participants increase the speed of their hand movements, and with decreasing tempo they slow down. Musical training, however, significantly influences this effect, such that untrained participants do not show an increase in speed of hand movement when the tempo is accelerating but only a decrease in speed when the tempo is decelerating. While differences between musically trained and untrained participants pertaining to imagined speed have been reported before for stimuli varied in inter-onset intervals and articulation (Eitan and Granot, 2006), the present interaction effect between tempo and training presents a novel finding.

Crucially, pitch influences the association between tempo and speed too. While the direction of pitch has no influence on the association between decelerating tempo and decrease in speed, falling pitch inhibits increase in speed in response to accelerating tempo. Note that falling pitch—represented by a downward hand movement—paired with accelerating tempo manifests the prototypical *physical* prerequisites for accelerated movement: an object (here the hand) accelerating toward the ground. There is, however, no increase in speed, which could be explained by semantics taking precedence over gravity. If falling pitch is conceived of as LESS and accelerating tempo is conceived of as MORE, this might create a semantic conflict, preventing the speed of hand movement from increasing. Another explanation could be the sense of intensity that is felt when various musical parameters interact. When musical parameters are aligned (e.g., falling pitch and decreasing tempo), the resulting change in speed mirrors the feeling of intensity that is created by this alignment (e.g., decrease in speed). When musical parameters are opposed, however, the resulting change in speed (if any) is much harder to predict, as it depends on the salience of individual musical parameters which, in their sum, determine whether one feels the intensity increasing, decreasing or perhaps ambiguous.

Taken together, these findings substantiate not only evidence of the association between tempo and speed in bodily cross-modal mappings (Kohn and Eitan, 2009), but also provide new insights into how interactions of auditory features affect the resulting speed of the hand movement.

## General discussion

The findings from the present study provide further evidence that musical training is a factor influencing the consistency of cross-modal mappings. In line with previous research (Eitan and Granot, 2006; Rusconi et al., 2006), both pitch—particularly falling–rising pitch—and loudness are mapped more consistently by musically trained participants. It needs to be tested to what extent sensorimotor skills play a role here (Küssner and Leech-Wilkinson, 2014) and how auditory, tactile and motor perception interact when mapping sound features cross-modally in real-time. As this might depend on the spatial features of a certain instrument, it will be worthwhile comparing groups of different instrumentalists such as pianists and clarinetists in future experiments. What is more, musical notation might play a crucial role here, too, and it would be very valuable to compare cross-modal mappings of musicians who use notations with those who do not.

One recurring finding of the present study is the preference for convex shapes (increasing–decreasing contours). Although this effect was hypothesized for pitch mappings based on previous findings (Kohn and Eitan, 2009), its pervasiveness in other mappings (e.g., of loudness) and more complex interactions between musical parameters suggests a prominent role in gestural cross-modal mappings. Drawing on findings from dance and movement therapy, Kestenberg-Amighi et al. (1999 as discussed in Eitan, 2013a) propose a general preference for inverted U-shape contours based on the natural tendency of the body—and its various functions, e.g., respiration, heart rate—to grow first before shrinking. Moreover, Kohn and Eitan (2009) remind us that “rise before fall” is also a commonly observed pattern in music that has been widely discussed in musicology. For instance, analysing a large database of Western folk songs, Huron (1996) showed that convex melodic shapes are much more common than any other melodic contour, and Leech-Wilkinson (in press) recently discussed the role of increasing and decreasing intensities (“feeling shapes”), drawing on Stern's psychoanalytic theory of Forms of Vitality (Stern, 2010). Although speculative, this might reflect the fact that intensifying stimulus features are more salient than attenuating ones because they are more significant in the environment: an object accelerating poses a greater potential threat than an object that decelerates (see Neuhoff, 2001, for a discussion of the adaptive value of changes in loudness). Thus, increasing stimulus properties in any sensory modality—higher, louder, brighter, warmer—imply the approach of a potentially harmful object, raising an organism's attention and alertness.

There are a few limitations which need to be considered when interpreting the current dataset and designing future studies. Generally, one needs to be conscious of the nature of the cross-modal mappings measured experimentally—whether spontaneous or, as it were, mandatory—since apart from the paradigm itself, the instruction may crucially influence what is being measured (Rusconi et al., 2006). We chose the expression “represent sound gesturally” over instructions emphasizing a more communicative aspect of the gestures, e.g., “while listening to the music, move to it in an appropriate way, such that another child could recognize the music while watching your movements without sound” (Kohn and Eitan, 2009) or, pertaining to sound drawings, asking participants to “represent the sound on paper in such a way that if another member of their community saw their marks they should be able to connect them with the sound” (Athanasopoulos and Moran, 2013). Although constituting seemingly negligible differences in instruction, the resulting drawings and gestures may give rise to different outcomes, particularly in a cross-cultural context as discussed by Eitan (2013b).

What is more, the design of the stimuli needs attention. First, it should be acknowledged that tempo variations were not completely systematized to avoid an exponential increase in experimental stimuli: when the tempo was changed it consisted either of two decelerandi or two accelerandi, but never of a mixture of both tempi. Secondly, when several auditory features were varied concurrently, change of direction always happened at the same time after 4 s. Needless to say, in musical performances there can be all sorts of overlaps (e.g., a slow crescendo over several rising–falling pitch glides including a short decelerando at the end), creating a complex interplay of increasing and decreasing intensities that our set of pure tones is unable to match. And while our stimuli could have been much more complex to come closer to real musical stimuli, they could have included simpler variations as well—e.g., a single pitch ascent with concurrently varied loudness or tempo—to study the basic gestural mappings in more details. Thus, there is scope for future studies to investigate both ends of the spectrum. Thirdly, and perhaps most crucially, when varying pitch, loudness and tempo concurrently, the variations of individual sound features might be differentially salient. That is, it matters whether the pitch range encompasses half an octave or four octaves, or whether the change in loudness occurs over 80 or 10% of the maximum amplitude. It is therefore not implausible that pitch—not loudness—was represented on the y-axis because it was perceptually more salient. Had the pitch range only included four semitones (or had it been in a different register) and the change in loudness been made more extreme, it might well have resulted in loudness vs. height associations. Researchers thus need to take great care when designing auditory stimuli that are varied in several sound features.

Finally, it should be pointed out that the findings presented here do not capture the unique ways in which participants might have represented sound gesturally, not only because the applied motion capture system is insensitive to fine-grained hand movements but also because participants might have used—consciously or subconsciously—other parts of their bodies to represent the sound. While the focus here was on averaged responses of hand movements to get insight into a largely under-researched field, the role of fine-grained movements of hands, fingers and other body parts provides a fruitful path to explore in future studies.

## Conclusion and implications

In the present study we investigated gestural representations of pitch, loudness and tempo, providing a solid empirical basis for future studies concerned with bodily cross-modal mappings. We were able to show that musical training plays an important role in shaping bodily cross-modal mappings, e.g., giving rise to more consistent mappings and annulling the commonly observed bias for convex shapes. Loudness vs. distance associations appear to be less relevant if the opportunity is provided to link loudness to energy levels, which can be seen as the fundamental physical factor influencing amplitude (i.e., deflection of air molecules). Moreover, concurrently varied musical parameters have a significant effect on the ways in which people represent sound gesturally: interactions between pitch and loudness affect how participants adjust the speed of their hand movement. Recent theoretical refinements of action-perception couplings in music perception provide an adequate framework in which such interaction effects may be investigated further (Maes et al., 2014). While it remains to be seen what the underlying mechanisms (e.g., perceptual, semantic) of these bodily cross-modal mappings are, the findings provided here may provide further support for the existence of recently developed concepts within embodied music cognition such as Godøy's (2006) “gestural-sonorous objects,” emphasizing the interconnection of motion and sound features in the mind of the listener. Facilitated by advances in multimedia technology (Tan et al., 2013) and the development of new musical instruments, the increasingly complex role of movement in creating and manipulating sounds and music challenges findings of cross-modal correspondences that have been obtained with traditional paradigms. Future studies need to address whether findings from bodily cross-modal mappings can be integrated wholly into current theoretical frameworks or whether “embodied cross-modal correspondences” might form a separate category worth studying in its own right. Besides theoretical implications, the outcome of the present study, as well as its low-cost motion capture devices, may be used in clinical settings where sounds and music are used to co-ordinate movement. For instance, music-based movement therapy has been found to be effective in treating Parkinson's disease (Rochester et al., 2010; De Dreu et al., 2012), and therapeutic approaches to stroke may benefit from musical activities, as shown in a study using the Wii™ Remote Controller to develop new forms of interventions (Van Wijck et al., 2012).

### Conflict of interest statement

The authors declare that the research was conducted in the absence of any commercial or financial relationships that could be construed as a potential conflict of interest.
